# Apolipoprotein A5 and apolipoprotein C3 single nucleotide polymorphisms are correlated with an increased risk of coronary heart disease: a case–control and meta-analysis study

**DOI:** 10.1186/s12944-015-0110-6

**Published:** 2015-09-19

**Authors:** Guang Yang, Ming-Ming Lei, Chun-Lei Yu, Xiao-Xiao Liu, Zhe An, Chun-Li Song

**Affiliations:** Department of Molecular Biology, College of Basic Medical Science, Jilin University, Changchun, 130000 P.R. China; Department of Internal Medicine-Cardiovascular, the Fourth Affiliated Hospital of China Medical University, Changchun, 130000 P.R. China; Department of Neurosurgery, FAW General Hospital, Jilin University, Changchun, 130000 P.R. China; Department of Cardiology, China-Japan Union Hospital, Jilin University, No. 126 Xiantai Stree, Changchun, 130000 P.R. China

**Keywords:** Apolipoprotein A5, Apolipoprotein C3, Single nucleotide polymorphism, Coronary heart disease, Triglyceride

## Abstract

**Background:**

Triglycerides (TGs) are proatherogenic lipoproteins involving the risk of coronary heart disease (CHD), while apolipoprotein A5 (APOA5) and apolipoprotein C3 (APOC3) are main lipoproteins composing TG-rich lipoproteins. In this study, we aim to explore the correlation of CHD with *APOA5* -1131 T > C and *APOC3* -455 T > C single nucleotide polymorphisms (SNPs).

**Methods:**

A sum of 210 CHD patients, hospitalized between Jan. 2013 and Mar. 2015 at China-Japan Union Hospital, Jilin University, were selected as our case group and 223 healthy individuals who had physical examination at same hospital at the same period were selected as control group. The frequency distribution of genotypes of *APOA5* -1131 T > C and *APOC3* -455 T > C SNPs were measured by polymerase chain reaction-restriction fragment length polymorphism (PCR-RFLP). The Stata 12.0 software was utilized for statistical analyses.

**Results:**

There was no significant difference on age and sex between case and control group (*P* > 0.05). History of smoking, drinking, hypertension and diabetes mellitus, body mass index and levels of TG and fasting blood sugar in case group were shown to be higher than control group (*P* < 0.05), while levels of total cholesterol, high-density lipoprotein cholesterol and low-density lipoprotein cholesterol in case group were lower than control group (*P* < 0.05). Both CC and TC′ + CC frequencies of *APOA5* -1131 T > C and *APOC3* -455 T > C in case group were higher compared to control group (both *P* < 0.05). Additionally, T allele frequencies of the two SNPs in case group were lower than control group, while C allele in case group has higher frequencies compared to control group (both *P* < 0.05). The results of meta-analysis under allele and dominant models showed that *APOA5* -1131 T > C and *APOC3* -455 T > C SNPs are likely to increase the risk of CHD (both *P* < 0.05).

**Conclusion:**

*APOA5* -1131 T > C and *APOC3* -455 T > C SNPs may play potent roles in the development and progression of CHD.

## Introduction

Coronary heart disease (CHD), also known as coronary atherosclerotic heart disease is a kind of thrombotic arterial disease, caused by hypoxia and myocardial ischemia owing to coronary atherosclerotic mediated vascular obstruction as well as coronary functional change [1, 2]. CHD has become one of leading diseases threatening human health and causing mortality, with an estimated 7.2 million individual worldwide die of this disease annually based on the World Health Organization [3]. In 2015, CHD alone caused approximately one of every seven deaths in the United States, and each year, there has emerged about 635,000 Americans who have a new coronary attack [4]. CHD is a complex multifactorial disorder and both non-genetic and genetic factors can contribute to the development and progression of this disease [5]. The non-genetic factors attributed to CHD has been clearly established, including type 2 diabetes, obesity, dyslipidemia, hypertension, low-density lipoprotein cholesterol (LDL-C), high density lipoprotein cholesterol (HDL-C) and lifestyle, such as smoking, lack of exercise and high fat diet [6, 7]. Interestingly, there is an inverse relationship between triglyceride (TG) levels and HDL-C levels, and in metabolic syndrome, high LDL levels reduce the TG content of HDL, thus reducing the size of the HDL particle and making it more prone for renal clearance; besides, excess of TG in the HDL particle contribute to HDL dysfunction [8]. Circulating triglyceride-rich lipoproteins from hypertriglyceridemic subjects mediated endothelial inflammation and drive monocyte infiltration into the wall of arterial [9]. Although prime interest was devoted to LDL-C as proatherogenic, TG are considered in the last years to be also proatherogenic and some studies provide evidence for causal involvement of TG-mediated pathways in CHD and the concentration of it (both fasting and non-fasting) in prediction of future cardiovascular disease events [9–14].

Apolipoprotein A5 (APOA5) is a fourthly discovered member of the APOA4/APOC3/APOA1 apolipoprotein cluster and the human *APOA5* gene is located at chromosome 11q23 [15]. *APOA5* gene comprises four exons and encodes APOA5, a protein of 366 amino acids, which is an effective stimulator of lipoprotein lipase and can facilitate lipoprotein remnant clearance in a LDL receptor-dependent manner [16, 17]. It was reported that nucleotide sequence variations in the *APOA5* gene have been correlated to high TG levels, exerting pleiotropic influences on different groups [18]. Apolipoprotein C3 (APOC3) is a significant component of TG-rich lipoproteins and a minor component of HDL [19]. *APOC3* gene, also located in the chromosome 11q23, participates in transport and clearance of very-low-density lipoprotein (VLDL), chylomicron remnants, and HDL from the bloodstream [20]. *APOC3* encodes a 79-amino-acid glycoprotein, which was produced largely in the liver interfering with receptor induced lipoprotein uptake and inhibiting the activation of lipoprotein lipase [21]. An increasing number of evidence suggested that *APOA5* -1131 T > C and *APOC3* -455 T > C single nucleotide polymorphisms (SNPs) contribute a substantial role in development of CHD owing to their correlation with increased plasma TGs, which has become the focus of oversea and domestic researchers [22, 23]. Nevertheless, there also emerged contradictory results on the role of *APOA5* and *APOC3* variants in CHD [24, 25]. In consideration of the controversial results from previous studies, we performed a case–control study to clearly address the correlation of CHD with *APOA5* -1131 T > C and *APOC3* -455 T > C, which was further confirmed by a following meta-analysis.

## Materials and methods

### Subjects

A total of 210 CHD patients (141 male and 69 female), hospitalized between Jan. 2013 and Mar. 2015 at China-Japan Union Hospital, Jilin University, were selected as our case group, among which 70 were acute myocardial infarction (MI), 109 were angina pectoris (27 stable, 82 unstable) and 31 were old MI. All CHD patients aged from 47 to 80 years, with mean age of 62.76 ± 9.98 years, and their diagnoses were based on American College of Cardiology/American Heart Association (2013) [26]. The diagnosis criteria were at least one with a diameter stenosis of ≥ 50 % in left main, left anterior descending, left circumflex and right coronary arteries, further examined by coronary arteriography and then evaluated through two interventional cardiologists. In addition, 223 healthy individuals (139 males and 84 females) who had physical examination at China-Japan Union Hospital, Jilin University at the same period were selected as control group, aged from 46 to 81 years (mean age: 62.44 ± 10.16 years). All subjects in control group had no positive sign, without history of CHD, cerebrovascular diseases or peripheral vascular diseases, and they showed normal in routine screening of blood and urine, chest X-ray, and liver and kidney function. Subjects included in our study have no blood relationship each other and we excluded subjects who had acute inflammation, rheumaimmune systemic diseases, malignant tumors, liver and renal diseases and thyroid disease (except hypertension and diabetes mellitus), and took lipid-lowering drugs in nearly four weeks. This study was authorized by the Ethical Committee of China-Japan Union Hospital, Jilin University, and all subjects included in our study provided written informed consent. All procedures in this study were in compliance with the Declaration of Helsinki [27].

### Determination of biochemical indexes

Peripheral blood (5 ml) was collected from each subject on an empty stomach for 12 h. OLYMPUS AU640 Analyzer (YZB/JAP 0357) was employed to determine levels of TG, total cholesterol (TC), HDL-C, LDL-C and fasting blood sugar (FBS).

### SNPs detection

**Fig. 1:**
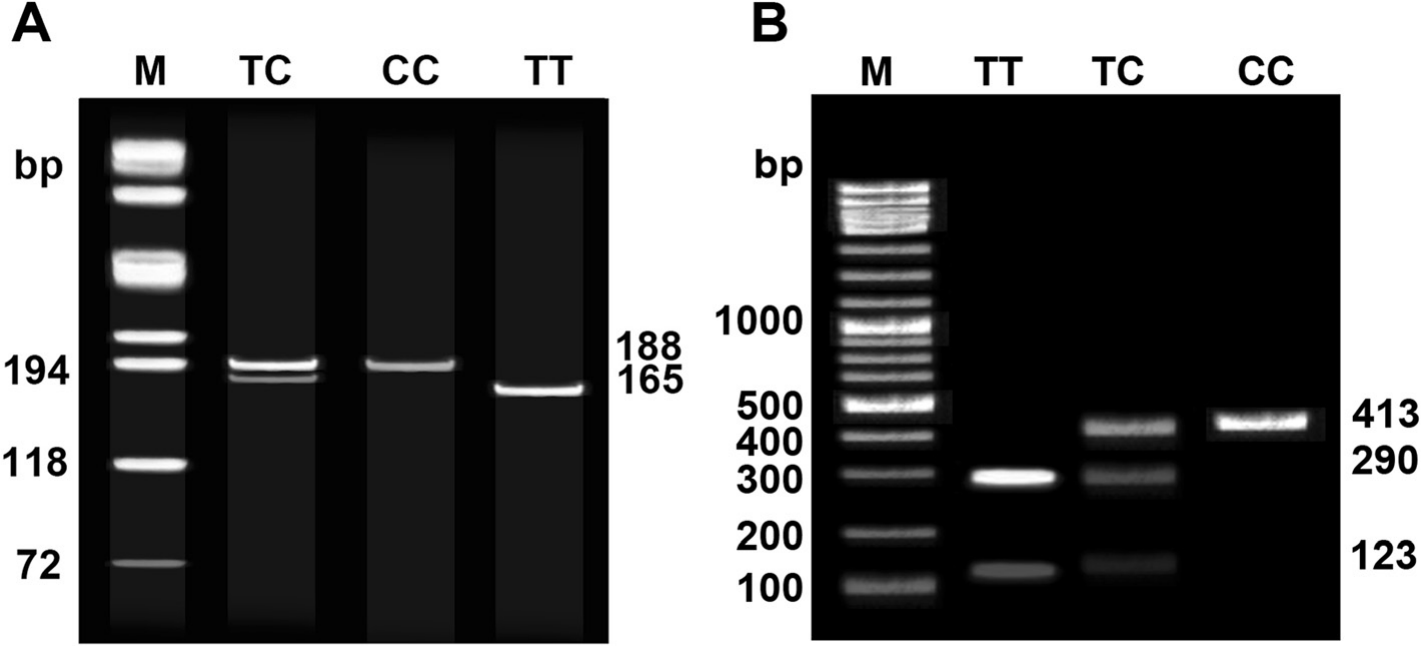
Gel electrophoresis of apolipoprotein A5 (*APOA5*) -1131 T > C (rs662799) and apolipoprotein C3 (*APOC3*) -455 T > C (rs2854116) single nucleotide polymorphisms (SNPs) after enzyme digestion (**a**: -1131 T > C (rs662799) of *APOA5*; **b**: -455 T > C (rs2854116) of *APOC3*)

*APOA5* rs662799 and *APOC3* rs2854116 SNPs were selected as our research targets. DNA extraction was undergone in the silicone membrane genomic DNA purification kit (Beijing SBS Genetech Co., Ltd.). The frequency distribution of allele and genotype of different SNPs in case and control group were measured by polymerase chain reaction-restriction fragment length polymorphism (PCR-RFLP). PCR primers were designed by Primer Premier 5.0 software (PREMIER Biosoft International, USA) and synthesized by Beijing Aoke Biotechnology Co., Ltd, and amplification site, primer sequence, fragment length, annealing temperature and number of cycles were presented in Table 1. For *APOA5* -1131 T > C (rs662799), PCR amplification reaction was performed in a 20 μl reaction containing DNA 40 ng, 0.2 mol/L of each forward and reverse primer, 200 μmol/L dATP, dGTP, dCTP and dTTP, 0.5U Taq DNA polymerase and PCR buffer solution. PCR reaction was performed under the following conditions: predenaturation at 96 °C for 5 min with a total of 35 cycles, denaturation at 96 °C for 45 s, annealing at 59 °C for 30s and elongation at 72 °C for 45 s, with lastly elongation at 72 °C for 7 min. The PCR amplification products were allowed to digest with Mse I restriction enzyme (MBI Company) overnight, and digestion products were subjected to 10 % polyacrylamide gel electrophoresis. The gel was visualized by ethidium bromide staining and then photographed under ultraviolet (UV) light. With regard to *APOC3* -455 T > C (rs2854116), PCR reaction mixture (50 μl) contains 10 × buffer 5 μl, dNTP 200 μmol/L, forward primer 25 pmol, reverse primer 25 pmol, genome DNA 50 ng and Taq DNA polymerase 0.5 U. After annealing at 60 °C for 30 cycles, 10 μl of PCR amplification products were digested at 37 °C by 8 U BseG I, from which 2 % gel electrophoresis was utilized to identify genotype, and the results were examined under UV light and then photographed. Gel electrophoresis of *APOA5* -1131 T > C and *APOC3* -455 T > C were shown in Fig. 1a ~ b.

**Table 1:** Primer sequencing of *ApolipoproteinA5* (*APOA5*) -1131 T > C and *apolipoproteinC3* (*APOC3*) -455 T > C single nucleotide polymorphisms (SNPs)

SNP	Primer	Fragment length (bp)	Annealing (°C)	Cycles
−1131 T > C (rs662799)	F: 5′-GATTGATTCAAGATGCATTTAGGAC-3′	188	59	35
	R: 5′-CCCCAGGAACTGGAGCGAAATT-3′			
−455 T > C (rs2854116)	F: 5′- GCACTCGCCTGCCTGGATT-3′	413	60	35
	R: 5′- TGATGCCACGCTGCTGTCCC-3′			

*F* forward, *R* reverse, *SNP* single nucleotide polymorphism

### Statistical analysis

Data analyses of our case–control study were conducted using SPSS 18.0 software (IBM Corporation, Somers, NY, USA). Distributions of continuous variables were presented as means ± standard deviation, which was further examined with the use of *t* test, while quantitative data were shown as percent, which also further analyzed by chi-square test. In order to analyze deviation from the Hardy-Weinberg equilibrium, the observed and expected frequencies of genotype were compared with the utilization of chi-square test. Odds ratio (OR) with 95 % confidence interval (CI) were calculated to evaluate differences of genotype and allele frequencies between case and control groups. Two-tailed *P* values of < 0.05 were statistically significant. With regard to data analyses in the following meta-analysis, STATA 12.0 software (Stata Corp, College Station, TX, USA) was used. OR with 95 % CI in fixed-effect model or random-effect model, presented in forest plot, was calculated to assess the strength of the association of CHD with *APOA5* -1131 T > C and *APOC3* -455 T > C, whose overall estimates were examined by *Z* test [28].

## Results

### Clinical data and biochemical index

There was no significant difference on age and sex between case and control group (*P* > 0.05). History of smoking, drinking, hypertension and diabetes mellitus, and levels of TG and FBS, and body mass index (BMI) in case group were shown to be higher than control group (*P* < 0.05), while levels of TC, HDL-C and LDL-C in case group were lower than control group (*P* < 0.05) (Table 2).

**Table 2:** Comparison of clinical data and biochemical index between case and control groups (mean ± standard deviation)

Parameters	Case group (*n* = 210)	Control group (*n* = 223)	*P*
Mean age	62.76 ± 9.98	62.44 ± 10.16	0.7413
Sex (n, %)			
M	141 (67.1 %)	139 (62.3 %)	0.2952
F	69 (32.9 %)	84 (37.7 %)	
Smoking status (n, %)			
Yes	113 (53.8 %)	61 (27.4 %)	< 0.0001
No	97 (46.2 %)	162 (72.6 %)	
Drinking status (n, %)			
Yes	128 (61.0 %)	93 (41.7 %)	< 0.0001
No	82 (39.0 %)	130 (58.3 %)	
Hypertension status (n, %)			
Yes	140 (66.7 %)	70 (31.4 %)	< 0.0001
No	70 (33.3 %)	153 (68.6 %)	
Diabetes mellitus (n, %)			
Yes	36 (17.1 %)	8 (3.6 %)	< 0.0001
No	174 (82.9 %)	215 (96.4 %)	
TG (mmol/L)	2.41 ± 1.33	1.64 ± 1.00	< 0.0001
TC (mmol/L)	4.74 ± 0.98	5.05 ± 0.89	0.0006
HDL-C (mmol/L)	0.97 ± 0.32	1.33 ± 0.32	< 0.0001
LDL-C (mmol/L)	2.81 ± 0.76	3.07 ± 0.75	0.0004
FBS (mmol/L)	6.30 ± 1.60	5.08 ± 0.62	< 0.0001
BMI (kg/m^2^)	25.77 ± 3.21	24.29 ± 2.72	< 0.0001

*M* male, *F* female, *TG* triglyceride, *TC* total cholesterol, *HDL-C* high-density lipoprotein cholesterol, *LDL-C* low-density lipoprotein cholesterol, *FBS* fasting blood sugar, *BMI* body mass index

### Distribution of *APOA5* -1131 T > C and *APOC3* -455 T > C SNPs

Distribution frequencies of genotypes of both *APOA5* -1131 T > C and *APOC3* -455 T > C SNPs complied with Hardy-Weinberg equilibrium in case and control groups (all *P* > 0.05), which indicated that this two SNPs were shown to be representative in this population. As shown in Table 3, both CC and TC′ + CC frequencies of *APOA5* -1131 T > C and *APOC3* -455 T > C in case group were higher compared to control group (both *P* < 0.05). Additionally, T allele frequencies of the two SNPs in case group were lower than control group, while C allele in case group has higher frequencies compared to control group (both *P* < 0.05).

**Table 3:** Distribution frequencies of genotypes in both *apolipoproteinA5* (*APOA5*) -1131 T > C and *apolipoproteinC3* (*APOC3*) -455 T > C single nucleotide polymorphisms (SNPs) between case and control groups

SNP	Control group (*n* = 223)	Case group (*n* = 210)	*P*	OR	95 % CI
−1131 T > C (rs662799)					
TT	96 (43.0 %)	69 (32.9 %)	Ref		
TC	107 (48.0 %)	101 (48.1 %)	0.194	1.313	0.870 ~ 1.983
CC	20 (9.0 %)	40 (19.0 %)	0.001	2.783	1.497 ~ 5.171
TT	96 (43.0 %)	69 (32.9 %)	Ref		
TC + CC	127 (57.0 %)	141 (67.1 %)	0.029	1.545	1.044 ~ 2.285
T	299 (67.0 %)	239 (56.9 %)	Ref		
C	147 (33.0 %)	181 (43.1 %)	0.002	1.54	1.169 ~ 2.031
−455 T > C (rs2854116)					
TT	102 (45.7 %)	75 (35.7 %)	Ref		
TC	101 (45.3 %)	97 (46.2 %)	0.199	1.306	0.868 ~ 1.965
CC	20 (9.0 %)	38 (18.1 %)	0.002	2.584	1.392 ~ 4.795
TT	102 (45.7 %)	75 (35.7 %)	Ref		
TC + CC	121 (54.3 %)	135 (64.3 %)	0.034	1.517	1.031 ~ 2.233
T	305 (68.4 %)	247 (58.8 %)	Ref		
C	141 (31.6 %)	173 (41.2 %)	0.003	1.515	1.147 ~ 2.002

*SNP* single nucleotide polymorphis

### Meta-analysis results

**Fig. 2:**
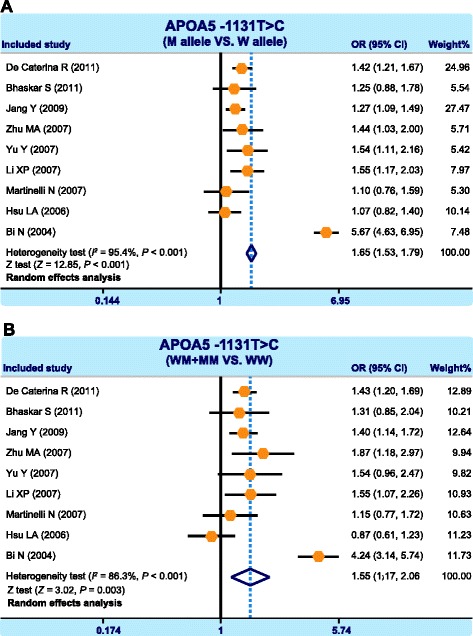
Forest plots showing the correlations between apolipoprotein A5 (*APOA5*) -1131 T > C (rs662799) single nucleotide polymorphisms (SNPs) and coronary heart disease (**a**: allele model; **b**: dominant model)

**Fig. 3:**
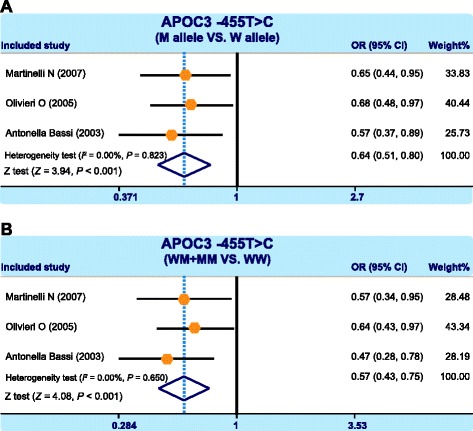
Forest plots showing the correlations between apolipoprotein C3 (*APOC3*) -455 T > C (rs2854116) single nucleotide polymorphisms (SNPs) and coronary heart disease (**a**: allele model; **b**: dominant model)

Our meta-analysis enrolled a total of 11 case–control study [24, 29–38] including 4840 CHD patients and 4913 health individuals. The results, pooled from 9 studies reporting the association between CHD and *APOA5* -1131 T > C, suggested that under allele model and dominant gene model -1131 T > C SNP is likely to increase the risk of CHD (allele model (C compared to T): OR = 1.65, 95 % CI = 1.53 ~ 1.79, *P* < 0.001, Fig. 2a; dominant model (TC+ CC compared to TT): OR = 1.55, 95 % CI = 1.17 ~ 2.06, *P* = 0.003, Fig. 2b). There were 3 studies that reported the association between CHD and *APOC3* -455 T > C and meta-analysis results indicated that under allele model and dominant gene model -455 T > C SNP had potential to increase the risk of CHD (allele: OR = 0.64, 95 % CI = 0.51 ~ 0.80, *P* < 0.001, Fig. 3a; dominant: OR = 0.57, 95 % CI = 0.43 ~ 0.75, *P* < 0.001, Fig. 3b).

## Discussion

The presented study evaluated the correlations of CHD with *APOA5* -1131 T > C and *APOC3* -455 T > C SNPs. Furthermore, in order to obtain a more conclusive and definitive estimation of the correlation of CHD with the two SNPs, our new case–control study combined with a meta-analysis pooled results from previously published studies were systematically carried out. The overall results of our study suggested that there was a strong link between the two SNPs, *APOA5* -1131 T > C and *APOC3* -455 T > C, with increased risk of CHD.

CHD, also known as coronary artery disease, is a multifactorial disorder, the risk of which was involved with some plasma lipids such as TC as well as TG, LDL-C, and HDL cholesterol (HDL-C), and lifestyles including smoking, alcohol consumption [39]. Additionally, both hypertension and diabetes mellitus were also involved in the development and progression of CHD [40]. In our study, we compared the clinical data and biochemical indexes of subjects between CHD patients and health individual, and the results demonstrated that CHD patients had longer history of smoking, drinking, hypertension along with diabetes mellitus, and lower levels of TC, HDL-C and LDL-C compared to health individuals.

One of our results in our study was that *APOA5* -1131 T > C SNP is likely to increase the risk of CHD, which was further demonstrated by the following meta-analysis. APOA5, encoding apolipoprotein AV, is expressed in the liver and circulates on very low density lipoproteins, chylomicrons CM and HDL [41]. Apolipoprotein AV participates in the catabolism of triglyceride-rich particles by exerting the activation of lipoprotein lipase, leading to the promotion of TG hydrolysis [29]. The rare haplotype in vivo would result in the reduction of apolipoprotein AV synthesis, and thus there was less apolipoprotein AV that was available for incorporation into triglyceride-rich particles, with the consequence of less circulating apolipoprotein AV to promote the activation of lipoprotein lipase or to affect its receptor-mediated clearance, eventually developing atherosclerosis [10, 42]. In our study, CC and TC + CC frequencies of *APOA5* -1131 T > C were higher compared to health individuals. Consist with our results, Sarwar et al. reported that the -1131 T > C (rs662799) promoter polymorphism of the *APOA5* gene was related to higher VLDL concentration as well as smaller HDL particle size pathways by which triglyceride could exert influence on the risk of CHD [10].

Another results in the current study suggested that *APOC3* -455 T > C had potential to increase the risk of CHD, which also further demonstrated by our meta-analysis. The metabolism of circulating particles rich in TGs is closely influenced by their content in APOC3 encoded by *APOC3* gene, and it is a component that interferes with the lipoprotein lipase-triggered hydrolysis of these particles [21]. The *APOC3* gene is transcriptionally down-regulated through insulin levels, and sequences in the promoter region with high adherence for the nuclear transcription factors inducing the insulin response are highly polymorphic [43]. Variants at positions −455 in *APOC3* gene have been shown to have a decreased affinity for the nuclear transcription factors inducing the response, which is also relevant to a half of insulin-generated downregulation of *APOC3* gene expression [44]. APOC3 is an important inhibitor of the lipolysis of triglyceride-rich lipoproteins via countering APOC2 activation of lipoprotein lipase [45]. Interestingly, Hypertriglyceridemia has been accepted by the National Cholesterol Education Program Adult Treatment Panel III as a potent risk factor for the development and progression of CHD [46].

In conclusion, our study demonstrated that *APOA5* -1131 T > C and *APOC3* -455 T > C SNPs may play potent roles in the development and progression of CHD. On a more practical ground, further researches with larger sample size are needed to examine whether incorporation of the *APOA5* -1131 T > C and *APOC3* -455 T > C SNPs into a general algorithm including genetic and metabolic variables will help further improving the identification of individuals at early risk of CHD.
